# Diverse Ecological Strategies Are Encoded by *Streptococcus pneumoniae* Bacteriocin-Like Peptides

**DOI:** 10.1093/gbe/evw055

**Published:** 2016-03-15

**Authors:** Eric L. Miller, Monica I. Abrudan, Ian S. Roberts, Daniel E. Rozen

**Affiliations:** ^1^Faculty of Life Sciences, University of Manchester, Manchester, United Kingdom; ^2^Institute of Biology, University of Leiden, Leiden, The Netherlands

**Keywords:** bacteriocin, bioinformatics, ecological strategies, interference competition, antagonism

## Abstract

The opportunistic pathogen *Streptococcus pneumoniae* is commonly carried asymptomatically in the human nasopharynx. Due to high rates of cocolonization with other pneumococcus strains, intraspecific competitive interactions partly determine the carriage duration of strains and thereby their potential to cause disease. These interactions may be mediated by bacteriocins, such as the type IIb bacteriocins encoded by the *blp* (*b*acteriocin-*l*ike *p*eptide) locus. To understand *blp* diversity and evolution, we undertook a bioinformatic analysis of 4,418 pneumococcal genomes, including 168 newly sequenced genomes. We describe immense variation at all levels of genomic organization: Gene presence/absence, gene order, and allelic diversity. If we make the extreme and naive hypothesis that assumes all genes in this operon can assort randomly, this variation could lead to 10^15^ distinct bacteriocin-related phenotypes, each potentially representing a unique ecological strategy; however, we provide several explanations for why this extreme is not realized. Although rarefaction analysis indicates that the number of unique strategies is not saturated, even after sampling thousands of genomes, we show that the variation is neither unbounded nor random. We delimit three bacteriocin groups, which contain group-specific bacteriocins, immunity genes, and *blp* operon gene order, and argue that this organization places a constraint on realized ecological strategies. We additionally show that ecological strategy diversity is significantly constrained by pneumococcal phylogeny and clonal structure. By examining patterns of association between alleles within the *blp* operon, we show that bacteriocin genes, which were believed to function in pairs, can be found with a broad diversity of partner alleles and immunity genes; this overall lack of allelic fidelity likely contributes to the fluid structure of this operon. Our results clarify the diversity of antagonistic ecological strategies in the global pneumococcal population and highlight the potential role of *blp* bacteriocins in competition within the nasopharynx.

## Introduction

Approximately 14 million cases of pneumococcal diseases—including pneumonia, sepsis, and meningitis—are caused by the Gram-positive bacterium *Streptococcus pneumoniae* each year, resulting in an estimated 800,000 deaths per year in children under 5 years old (O’Brien et al. 2009). Additionally, up to 88% of children under 5 years old are asymptomatically colonized by *S. pneumoniae* in the nasopharynx (Regev-Yochay et al. 2004; Wyllie et al. 2014), which serves as a reservoir out of which strains migrate and cause disease (Bogaert et al. 2001). Pneumococcal conjugate vaccines have reduced childhood deaths by over 75% in areas where they are administered (Black et al. 2000; Klugman et al. 2003; O’Brien et al. 2003; Cutts et al. 2005). This vaccine has also resulted in a shift from vaccine serotypes to nonvaccine serotypes in the commensal pneumococcal populations that reside in the nasopharynx (Tocheva et al. 2011; Spijkerman et al. 2012; Davis et al. 2013). Vaccine-induced replacement, together with more advanced methods of detection, has led to an increased appreciation that simultaneous cocolonization with multiple pneumococcal strains is very common (Sauver et al. 2000; García-Rodríguez and Fresnadillo Martínez 2002; Brugger et al. 2010; Wyllie et al. 2014). Competitive interactions among these strains within the nasopharynx can therefore influence clonal frequencies, colonization dynamics, and, in turn, the potential for different bacterial strains to cause disease.

One of the key drivers of competitive dynamics between coexisting pneumococcal strains is small-peptide bacteriocins that regulate intraspecific killing. *Streptococcus pneumoniae* has at least four bacteriocin systems: The competence*-*regulated CibAB bacteriocin responsible for fratricidal killing (Guiral et al. 2005), the Phr lantibiotic (Hoover et al. 2015), the recently discovered pneumocyclicin (Bogaardt et al. 2015), and the *blp* (*b*acteriocin-*l*ike *p*eptides) operon (Dawid et al. 2007; Lux et al. 2007), which we focus on here. Regulation of the *blp* operon is coordinated by a typical Gram-positive quorum sensing two-component system (De Saizieu et al. 2000), with BlpH as a membrane-bound, histidine kinase receptor for the secreted quorum sensing signal peptide produced by *blpC* (De Saizieu et al. 2000; Reichmann and Hakenbeck 2000). When extracellular levels of BlpC surpass a threshold concentration, the peptide signal binds to BlpH (Pinchas et al. 2015), which activates the response regulator BlpR by phophorylation; this, in turn, increases production of the BlpC signal (De Saizieu et al. 2000) and activates putative bacteriocin genes *blpD*, *blpE*, *blpI*, *blpJ*, *blpK*, *blpM*, *blpN*, *blpO*, *blpW*, *pncT*, and *pncW* (Bogaardt et al. 2015). Before secretion, the N-terminal, double-glycine leader sequence of the translated signal and bacteriocins is cleaved, after which the mature peptide is exported by the ABC transporter system encoded by BlpA and BlpB (Håvarstein et al. 1995). *blp* bacteriocins are thought to be type IIb bacteriocins, which require equimolar production of two separate peptides to bind to the outside of a target cell to cause cell death (Nissen-Meyer et al. 2010). Notably, however, specific pairing has only been experimentally shown for a single pair of Blp bacteriocins, BlpM and BlpN (Dawid et al. 2007). Moreover, it remains unknown if pairs of two-peptide bacteriocins show absolute fidelity to one another, or if active bacteriocins can form between diverse peptide partners. To avoid bacteriocin-associated suicide as well as to defend against the bacteriocins of competing strains (Bogaardt et al. 2015), putative immunity genes *blpF*, *blpG*, *blpL*, *blpP*, *blpX*, *blpY*, *blpZ*, *pncG*, *pncM*, and *pncP* are also activated by the response regulator BlpR. Two additional genes, *blpT* and *blpS*, have an unknown role in the regulation of the *blp* operon (De Saizieu et al. 2000).

Although the general structure of the *blp* operon appears to be conserved among pneumococcal strains (Dawid et al. 2007; Lux et al. 2007), the specific composition of the operon is markedly variable (Bogaardt et al. 2015). First, the signal peptide pheromone of each strain, encoded by *blpC*, can vary. At least four distinct peptide types in the species have been described, each believed to bind most tightly to its specific cognate receptor (Pinchas et al. 2015), and here we describe the discovery of several additional potential signal types. Second, the set of bacteriocins carried by each strain can differ, indicating that there is variation in the chemical arsenal that each strain carries (Lux et al. 2007). Finally, strains vary in the number and type of immunity genes within this operon, implying between-strain differences in susceptibility (Lux et al. 2007). The various permutations of these components can be thought of as a vast ecological strategy set. Each strain from this set, with a unique combination of signal, killing, and immunity, thus expresses one ecological strategy that defines the fraction of potential competitors a given strain can kill, and the fraction to which it is susceptible. How many such strategies exist from among those that are possible? At present, the answer to this fundamental question remains unknown.

Using more than 4,000 publically available *S. pneumoniae* genomes together with 168 genomes sequenced for this study, we sought to answer this question using a bioinformatics approach. We ask the following specific questions: First, how diverse are *blp* signals, receptors, bacteriocins, and immunity genes, and what is the combinatorial complexity of this operon? Second, what subset of the combinatorial possibilities among these ecological strategies is actually observed? Is the realized strategy set biased to particular clonal complexes or combinations of alleles, implying that there are phylogenetic or functional constraints on operon structure? Finally, what is the correlational structure of signal, receptor, bacteriocin, and immunity genes? In brief, we identify unprecedented diversity in the pneumococcal *blp* operon with a combinatorial ecological strategy potential into the trillions. Yet despite this vast set of possibilities, the number of realized ecological strategies of signaling, bacteriocins, and immunity is significantly smaller. We discuss these results in the context of bacterial competitiveness and colonization dynamics within the human nasopharynx.

## Materials and Methods

### Genomes and Assemblies

We used *S. pneumoniae* genomic information from five publicly available data sets. This included the following: 297 fully assembled genomes from GenBank and the Sanger Institute FTP site, which included 121 assembled genomes from Georgia, United States (Chancey et al. 2015); 3,085 assembled contigs from Myanmar refugees (Maela data set; Chewapreecha et al. 2014); sequence reads for 616 carriage strains from Massachusetts (Croucher, Finkelstein, et al. 2013); sequence reads for 82 Complex 3 strains (Croucher, Mitchell, et al. 2013); and sequence reads for 242 PMEN-1 (Pneumococcal Molecular Epidemiology Network) strains (Croucher et al. 2011). Additionally, we extracted DNA and sequenced 142 carriage strains from The Netherlands (Hermans genome set; Bogaert et al. 2001) and 26 PMEN strains (McGee et al. 2001) using Hiseq Illumina sequencing (Leiden Genome Technology Center). These reads have been submitted to the European Nucleotide Archive as study PRJEB10892 and PRJEB10893 (Hermans genome set and PMEN strains respectively; accession numbers in supplementary table S1, Supplementary Material online).

For genome sets with sequence read data (the Massachusetts, Complex 3, PMEN-1, Hermans, and PMEN strains), we assembled sequence reads into contigs de nova using only unique sequence reads with no ambiguous bases and minimum Phred quality scores of 25, 35, and 45. Experimentally, we found that too many reads can interfere with the final quality of assembled genomes (data not shown), so we assembled genomes using a range of sequence reads that started at 1 million sequence reads, ended at the maximum number of sequence reads for each genome, and increased by intervals of 200,000 sequence reads. We selected the assembly with the highest N50 for each genome out of the assembly results. We used Velvet 1.2.10 (Zerbino and Birney 2008) and VelvetOptimiser 2.2.5 (Gladman and Seemann 2008) with hash values from 45 to 61 by intervals of 4 to assemble the genomes. All sets of reads were treated as unpaired, even in the presence of paired sequence reads, due to ambiguity in the distance between paired sequence reads.

The assembled contigs from the Maela genome set, as available from the Sanger FTP site, contained extensive evidence of perfectly duplicated *blp* regions. To overcome this artifact, we broke the assembled contigs from each of the Maela assembled genomes into fragments of 150 bp that overlapped by 25 bp, and then we reassembled the genomes as described above. Genomes thus assembled showed no evidence for the duplications in the original assemblies.

### Algorithm for Identifying Homologs

We developed an iterative DNA reciprocal BLAST (Altschul et al. 1990) algorithm for finding alleles in genes of interest across draft, nonannotated genomes (supplementary fig. S1, Supplementary Material online). This algorithm used two databases: A genome database of all contigs from the assembled genomes, and a filter database initially consisting of the DNA sequence open reading frames (ORFs) of annotated and unknown genes in 25 well-annotated *S. pneumoniae* genomes (supplementary table S2, Supplementary Material online). All unique annotated sequences in the filter database for a query gene were used to create a BLAST queue. Each of these gene variants was BLASTed against the genome database in turn. Reported sequences with an *e*-value of less than 10.0 were then BLASTed back into the filter database. Although this was a lenient criterion, only protein-coding sequences with a top hit of the same query gene were reported, as the sequences were then reciprocal best-BLAST hits, and this always resulted in alleles that easily aligned. To check against the risk of excess leniency, we ran the same DNA reciprocal BLAST searches using a vastly reduced *e*-value of 10^−^^20^ for all putative *blp* bacteriocins. This analysis recovered identical alleles as with the higher *e*-value threshold. A recovered sequence was scored as an allele only if it satisfied the following criteria: 1) The contig on which it was found contained an in-frame stop codon before the sequence’s start codon or the contig on which it was found lacked an upstream, in-frame stop code, but the sequence was a full-length allele for the gene; 2) the sequence was followed by a stop codon; and 3) translation of the sequence was at least 20 residues long, with an overall length between 75% and 125% of the average allele for the given locus. Novel allele sequences were added to the filter database and to the BLAST queue. The BLAST queue was iteratively cycled through until no new reported sequences were found. This iterative process allowed the gene sequences to move further away in sequence space from the initial 25 well-annotated genomes while preserving the criterion of reciprocal best-BLAST hits. The algorithm was implemented using custom Python scripts.

### Curating the Genome Set

To eliminate poorly assembled or misattributed genomes, we used the DNA reciprocal BLAST algorithm to search for three housekeeping genes that should be in every *S. pneumoniae* genome: *groEL*, *gyrA*, and *rpoD*. We eliminated genomes that lacked two or more of these housekeeping genes (strains 6938_7#19 and 6972_5#3) and genomes with non-*Streptococcus* variants of these genes (56 genomes; supplementary table S1, Supplementary Material online), as determined by top BLAST hits in the GenBank database. We also eliminated strain 484-93 because of its poor assembly and strain 06_01_003MEF_uid198409, as it contained 7,124 called genes (compared with a normal range of 1,800–2,100 called genes).

In order to estimate the whole-genome phylogeny of the strains, each genome was aligned to the R6_uid57859 genome using the following method. We first divided each assembled genome into 50 bp fragments that overlapped by 10 bp, and then we reassembled each genome with R6_uid57859 as a reference genome using Stampy 1.0.23 (Lunter and Goodson 2011) with a substitution rate of 0.01. For an alignment, we excluded sites with gaps or “N”s in more than 0.5% of genomes, resulting in 1,444,122 remaining sites. We aligned these sites with 43 *Streptococcus* sp. viridans genomes (supplementary table S3, Supplementary Material online) as an outgroup. We conducted 30 maximum-likelihood phylogenetic tree searches with ExaML 3.0 (Kozlov et al. 2015) using this alignment with 15 random, unique starting trees, and 15 unique parsimonious trees (as determined by RAxML 8.2.4; Stamatakis 2006) with the GTRCAT model of evolution and scored each resulting tree with the GTR + Gamma model of evolution under ExaML 3.0. We present the tree with the highest likelihood score from these searches (ln(likelihood) of −32145034.7). We created 100 random nonparametric bootstraps using RAxML 8.2.4 (Stamatakis 2006), and we searched for the best tree with a single ExaML search for each bootstrap (Kozlov et al. 2015) using a single starting parsimonious tree for each bootstrap (Stamatakis 2006). We collapsed branches with less than 75% bootstrap support. Genomes that clustered with the outgroup instead of with the remaining *S. pneumoniae* genomes were excluded from further analysis (12 genomes; supplementary table S1, Supplementary Material online). Together, this resulted in a final set of 4,418 *S. pneumoniae* genomes (supplementary table S1, Supplementary Material online). Of these, we considered 4,096 strains to be randomly sampled from global populations (by excluding the Complex 3 and PMEN-1 strains); percentages of gene presence are only for these randomly sampled genomes.

### Locating Alleles within the Genome Set

We used the DNA reciprocal BLAST algorithm described above to locate alleles of the *blp* operon genes. As a starting set of *blp* operon genes, we searched for all *blp*- or *pnc*-annotated genes in the 25 well-annotated genomes (supplementary table S2, Supplementary Material online). To search for novel *blp* operon genes not present in these annotated genomes, we used the SEED server (Aziz et al. 2008) to find protein-encoding genes in the 4,418 genomes that 1) were located between two previously found *blp* operon genes, 2) were found less than 2,000 bp from a *blp* operon gene, and 3) did not have significant BLAST hits in GenBank for transposon-related genes. We then searched for any such *blp* operon genes using the DNA reciprocal BLAST algorithm. Three hundred seventy-one genomes contained *blpT* and *pncP* (which are the acknowledged ends of the *blp* operon; Bogaardt et al. 2015) on a single contig; additional *blp* operon genes that were not in any of these 371 randomly sampled genomes are calculated to occur at a frequency of 0.8% or lower in the global *S. pneumoniae* population (α = 0.05). We were unable to find *blpW* in the 4,418 genomes (Bogaardt et al. 2015).

In addition to *blp* operon genes, we additionally searched our genome set for *comA*, *comB*, and *comC* to directly compare these alleles with their paralogs *blpA*, *blpB*, and *blpC*, respectively. In order to assign strains to sequence types (ST) via the *S. pneumoniae* MLST database (September 24, 2015 data set; Jolley and Maiden 2010), we also identified the seven genes used for MLST sequence typing (*aroE*, *gdh/gdhA*, *gki/glkA*, *recP*, *spi/lepB*, *xpt*, and *ddl/ddlA*). We used eBURST v3 (Feil et al. 2004; Spratt et al. 2004) and the *S. pneumoniae* MLST database to assign ST to clonal complexes, where a clonal complex contained ST that shared at least six identical MLST alleles with at least one other ST within the clonal complex. Although 173 genomes (3.9%) could not be assigned to an ST due to missing sequence data, 47 of these genomes were unambiguously assigned to clonal complexes.

As evidence of the success of our DNA reciprocal BLAST algorithm, we found the seven MLST genes in 99.3–99.5% of genomes and *comABC* in 98.6–99.8% of genomes. This gave us confidence that we could detect genes at a discovery rate of at least 98.6%, which included incomplete genomes in our data set.

Finally, we briefly examined evidence for deteriorated and potentially active transposase sequences within the *blp* operon, which could possibly serve as foci for homologous recombination. We searched for transposase-like sequences by locating all annotated transposase genes in the 25 well-annotated genomes and using the DNA reciprocal BLAST algorithm. Importantly, this search did not require an ORF in the resulting sequences, as we were also interested in nonfunctional transposases. As a result, we did not iteratively search for new transposases after an initial search that used the starting transposase sequences.

The results of all searches were stored in a custom SQL database for easy querying.

### Systemizing Variation

To assign putative functions for the *blp* operon genes, we BLASTed the amino acid sequence of all recovered alleles on both the UniProtKB/Swiss-Prot database and the nonredundant GenBank protein sequence database. We classified eight genes with no significant hits and shorter than 40 residues as probable untranslated ORFs (supplementary table S4, Supplementary Material online).

We identified extensive amino acid variation in *blp* operon genes across these thousands of genomes. To focus on larger patterns, we classified alleles within each locus into highly similar groups called “phylotypes”; while not designed as functionally distinct alleles, this grouping of alleles is more conservative than using amino acid variants. Phylotypes were determined differently for genes with or without presumed leader peptides. Quorum sensing signals and presumed bacteriocin peptides contain short leader peptides that are typically cleaved following a conserved double-glycine sequence before the mature bacteriocin is exported by ABC transporters. All unique sequences past the double-glycine were classified as a different phylotype for the quorum sensing signal *blpC* and putative bacteriocins *blpD*, *blpE*, *blpI*, *blpI2*, *blpJ*, *blpK*, *blpM*, *blpN*, *blpO*, *blpQ*, *pncT*, and *pncW*. Notably, we identified four presumed, distinct leader sequences in *blp* operon genes that were classified as putative bacteriocin by protein BLAST searches (supplementary table S5, Supplementary Material online). These presumed leader sequences had a Glu/Met residue preceding a Leu six residues before the double-glycine cleavage site (ELSNISGG, MLSEVYGG, and MLAXVEGG); this motif was also found in the leader sequence for BlpC (ELNQITGG), whereas ComC had an Asp-Leu motif six residues before the cleavage site (DLQKIKGG)*.* In place of a double-glycine cleavage site, 13.9% of genomes had an Asp-Gly site specifically in PncW (MLAVRTEDG); as these residues were found in high frequency, it suggested the possibility of flexibility in the leader sequence cleavage site, assuming these sequences indeed permit mature peptide secretion. For genes without a leader sequence, we aligned protein variants for each locus and created a neighbor-joining tree using Geneious 7.1.5 (Kearse et al. 2012). The protein variants on these trees were impartially divided into phylotypes based on subclades using 3 rules: 1) Excluding branches with branch lengths over 3.5 standard deviations from the mean branch length; 2) excluding branches with branch lengths over 0.025; and 3) dividing clades so that the maximum intraclade distance was 0.05. For analysis, we created an arbitrary cut-off of 0.5% of the 4,096 randomly sampled genomes for “common” phylotypes to avoid focusing on singleton strains and their recently derived clone mates.

### Statistical Analyses

Type IIb *blp* bacteriocins are predicted to work as a pair of peptides that interact at equimolar concentrations to induce lethality to target cells. Each active “pair” is believed to associate with an immunity gene that prevents self-toxicity. It is presently unknown if peptide pairs that create active bacteriocins are specific with respect to their patterns of association, or if peptides can assort more broadly to generate an active toxin. Equally, it remains unknown if immunity is specific to certain bacteriocins or if there is cross-immunity, where individual immunity proteins provide more generalized protection. We used the following procedure to test for significant coassociation of genes while accounting for phylogeny. For each phylotype, we scored each genome as either 1) containing the phylotype, 2) containing the same gene but a different phylotype, or 3) not containing the gene. In the last case, the genome was treated as “missing data” either from having incomplete information from draft genomes or from a complete genome not containing the gene. Then, for all pairs of phylotypes that co-occur in at least 0.5% of genomes and that belong to different genes, we used BayesTraits 2.0 (Pagel et al. 2004) to estimate the coassociation between phylotypes. BayesTraits calculated the maximum likelihood of the phylotypes’ patterns along the phylogenetic tree assuming that the phylotypes mutated their presence/absence independently or assuming that the phylotypes influenced each other. We used the whole-genome phylogenetic tree with a conservative bootstrap threshold of 75% in order to examine only well-supported clades. We tested for significance using a log likelihood test and post hoc corrected for multiple tests within each pair of genes using a Holm-Bonferroni correction (Holm 1979). All other statistical analyses were performed using RStudio 0.98.507 (RStudio Team 2015) and R 3.1.3 (R Core Team 2013).

Nucleotide diversity and *d*_N_/*d*_S_ was measured from aligned alleles for each gene using DnaSP 5.10.01 (Librado and Rozas 2009). Analyses were performed on only amino acid coding regions. Aligned sites and whole sequences were removed if they contained indels found in less than 5% of the alleles.

## Results

### Diversity in the *blp* Operon

**Fig. 1.—:**
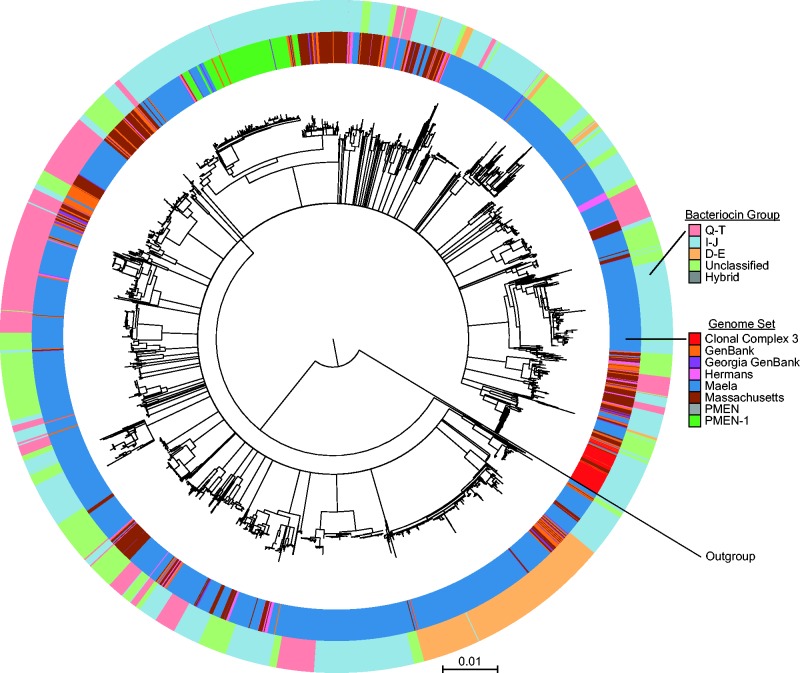
Phylogenetic relationship between 4,418 S*treptococcus* pneumoniae genomes. We used 43 nonpneumoniae *Streptococcus* sp. as an outgroup. We collapsed clades with less than 75% nonparametric bootstrap support. The colored rings show the genome set (inner ring) and bacteriocin group (outer ring) of each genome.

We investigated the diversity of the *blp* operon of 4,418 *S. pneumoniae* genomes across 5 publically available genome sets taken from around the world, together with 142 carriage strains isolated from healthy Dutch children (Hermans set, Bogaert et al. 2001) and 26 PMEN strains (McGee et al. 2001), which were sequenced for this study (fig. 1). Using a DNA reciprocal BLAST search (supplementary fig. S1, Supplementary Material online), we found 88,498 homologs of genes in the *blp* operon divided across 35 loci (table 1).

**Table 1:** Genes and Gene Frequencies of *blp* Operon and Related Genes

Gene	Alternative Names	Function^a^	Frequency^b^^,^^c^	Amino Acid Variants	Phylotypes over 0.5% Frequency^b^
*blpA*	*spiCBA*	ABC transporter	0.249	272	23
*blpB*	*spiD*	ABC transporter	0.838	195	11
*blpC*	*spiP*	QS signal	0.991	29	9
*blpD*	*blpM, blpO*	Bacteriocin^d^	0.115	4	1
*blpE*		Bacteriocin^d^	0.116	2	1
*blpF*		Membrane protein^d^	0.114	2	1
*blpG*		CAAX protease^d^	0.111	3	1
*blpH*	*spiH*	QS receptor	0.990	156	15
*blpI*	*pncA*	Bacteriocin^d^	0.404	11	3
*blpI2*		Bacteriocin^d^	0.002	1	0
*blpJ*	*pncD*	Bacteriocin	0.416	22	5
*blpK*	*pncE, thmA, blpU*	Bacteriocin	0.545	50	8
*blpL*	*pncH*	Membrane protein^d^	0.575	59	9
*blpM*	*pncI*	Bacteriocin	0.610	33	5
*blpN*	*pncJ, blpM, blpK*	Bacteriocin	0.757	26	6
*blpO*	*pncV, pncL*	Bacteriocin^d^	0.605	24	4
*blpP*	*pncK*	Membrane protein^d^	0.864	18	2
*blpQ*	*pncR*	Bacteriocin^d^	0.202	4	1
*blpR*	*spiR2*	Response regulator	0.995	91	7
*blpS*	*spiR1*	Accessory protein	0.938	70	9
*blpT*		Unknown	0.991	48	7
*blpU1*		Unknown	0.059	7	2
*blpU2*		Unknown	0.006	4	1
*blpU3*		Unknown	0.213	6	2
*blpU4*	*pncB*	Unknown	0.425	6	2
*blpU5*		Unknown	0.422	7	1
*blpV*		Unknown	0.116	2	1
*blpX*	*pncN*	Membrane protein^d^	0.692	28	4
*blpY*	*pncO*	CAAX protease^d^	0.983	96	10
*blpZ*	*pncQ*	Membrane protein^d^	0.993	43	9
*comA*		ABC transporter	0.983	181	5
*comB*		ABC transporter	0.992	121	2
*pncG*		Membrane protein^d^	0.792	26	10
*pncM*		Membrane protein^d^	0.633	18	4
*pncP*	SP0547	CAAX protease^d^	0.984	91	9
*pncT*		Bacteriocin^d^	0.213	7	1
*pncW*	*blpN, blpO*	Bacteriocin^d^	0.302	20	4

^a^CAAX proteases and membrane proteins are both considered immunity genes.

^b^In 4,096 randomly sampled genomes.

^c^Full length alleles only for *blpA* and *blpB*.

^d^Putative, based on sequence similarity.

Examining such a large number of genomes enabled us to identify six additional *blp* operon genes compared with previous studies (e.g., *blpI2* and *blpU1–blpU5*; Lux et al. 2007; Bogaardt et al. 2015). We also exhaustively characterized within-locus allelic diversity for all identified *blp* operon genes (table 1). Overall, we found between 1 and 272 amino acid variants in these genes (average = 42.3 amino acid variants; table 1). With one exception, all *blp* operon genes were confined to the *blp* locus. *blpK* was present in a single copy in 47.6% of genomes and duplicated in 4.9% of genomes; this gene was located either within the *blp* locus (6.9% of strains), adjacent to the competence peptide transporter *comAB* elsewhere in the genome (11.2% of strains), or in an undetermined location (41.1% of strains). No other *blp* operon gene (table 1) was found more than 16,341 bp (the length of the largest fully assembled *blp* operon, in strain Taiwan19F_14_uid59119) outside of the canonical operon delimited by *blpT* or *pncP* (in a single exception to this finding, *blpMNPO* is directly adjacent to *comAB* in strain 6259_8#1).

Confusingly, three separate nomenclatures (i.e., *blp*, *pnc*, and *spi*) are currently used for genes within this operon (Bogaardt et al. 2015). Given the comprehensive nature of our DNA reciprocal BLAST search and to avoid confusion here and elsewhere, we adopted the most recent and inclusive nomenclature from Bogaardt et al. (2015). To their list, we add an additional putative bacteriocin gene *blpI2*, which occurs at low frequency (less than 0.5%) with no similar protein BLAST hit, and five hypothetical genes *blpU1*–*blpU5* (tables 1 and 2). One *blpM* variant (distinct from *blpMN*; Bogaardt et al. 2015) in 1.1% of genomes encoded a hybrid bacteriocin containing BlpM and BlpN on a single reading frame, wherein BlpM was followed by a second leader sequence and BlpN without a stop codon between the two (table 2). It is possible that this structure would allow for production of two bacteriocin peptides from a single reading frame.

**Table 2:** Mature Putative Bacteriocin Amino Acid Sequences

Gene	Frequency in Randomly Sampled Genomes^a^	Amino Acid Sequence
*blpD*	0.115	TDWGTVGKGAVYGAGIGVAMCAVGGLLTGGSTWAMTAGCAWAGAKLGGSFTAIADNLWP
*blpE*	0.116	GLGGDVVVGALSGAFQAGQSCIAGGPQAYLICATGGAIVGGILAYGLRPPK
*blpI*	0.371	RGNLGSAIGGCIGAVLLAAATGPITGGAATLICVGSGIMSSL
	0.025	...............................T..........
	0.006	.......................................P..
*blpJ*	0.349	YSSTDCQNALITGVTTGIITGGTGAGLATLGVAGLAGAFVGAHIGAIGGGLTCLGGMVGDKLGLSW
	0.027	...............................................R..................
	0.017	..F...............................................................
	0.006	................................................S.................
	0.005	...................................V............D.................
*blpK*	0.139	GCNWGDFAKAGVGGGAARGLQLGIKTRTWQGAATGAVGGAILGGVAYAATCWW
	0.119	....................................A................
	0.099	................V...................A................
	0.067	................- - - - - - - - - - - - - - - - - - -.............
	0.050	...............V....................A................
	0.044	................V...................A................
	0.034	..........................G.........A................
	0.029	..............A...........G.........A................
*blpI2*	0.002	DKVGAGEVVQALGICTIGGAALGSVIPVVGTLAGGILGAQFCTAAWGAFRAS
*blpM*	0.300	KNNWQTNVLEGGGAAFGGWGLGTAICAASGVGAPFMGACGYIGAKFGVDLWAGVTGATGGF
	0.101	................................................A............
	0.096	................................................A.........S..
	0.092	........F...S...................................A............
	0.011	................................................A............QQKETC MNTYCNINETMLSEVYGG
*blpN*	0.374	NS- - - - - - - - GGAAVVAALGCAAGGVKYGRLLGPWGAAIGGIGGAVVCGYLAYTATS
	0.149	..- - - - - - - - ...................KI..........................
	0.101	KNNWQTNVLEGG .- - - -..F......................................
	0.057	..- - - - - - - - .......................L.......................
	0.043	GCNWGDFAKAGV ...................KI..........................
	0.011	..- - - - - - - - ...................KI........–-...............
*blpO*	0.547	DIDWGRKISCAAGVAYGAIDGCATTV
	0.028	..........T...............
	0.017	......................V...
	0.007	......E...................
*blpQ*	0.200	IFGVDDALFWAGLGYVAGSIVDTAIDDFTNQCRKNPHQWFCVRV
*pncT*	0.207	DDCFIGDIGCIGWGLLKSIGGMIKPAPYVPPVCIPKSSWNPAPPVPC
*pncW*	0.132	DVSDIYRGYANQRSPFASYPSILKNSGPFPVSGYCLRGYHDRGYIGAGFHLCGI
	0.126	............V...G...P..............P..................
	0.028	...G......Y.D...GP.................P...R.L............
	0.005	...G......Y.D...GP........D........P...R.L............

^a^For genes present in over 0.5% of genomes, only variants found in at least 0.5% of genomes are shown.

To simplify the analysis of allelic diversity (table 1), we grouped amino acid variants within individual genes into *phylotypes* (see Materials and Methods), focusing on the most common phylotypes occurring in at least 0.5% of the 4,096 genomes from randomly sampled strain collections (by excluding the Complex 3 and PMEN-1 genome sets; table 1). Even using this conservative measure to describe allelic diversity within genes, thousands of genomes were required to effectively sample global *blp* operon diversity, thus emphasizing the necessity of a large data set. We performed 10,000 bootstrap rarefactions on the 4,096 randomly sampled genomes, which revealed that variation in nonsingleton phylotypes was saturated at approximately 3,000 and 2,000 genomes for putative immunity and bacteriocin genes, respectively (supplementary fig. S2, Supplementary Material online). This gave us confidence that the vast majority of the total variation in global *S. pneumoniae* populations was included in our analyses.

To investigate the tempo of molecular evolution at this locus, we placed these genes into putative functional categories: Bacteriocin genes, immunity genes, *blp* regulatory genes, and *blp* genes with unknown function, as well as examined nine housekeeping genes outside of the *blp* operon (table 3). Although the mean rate of nucleotide substitution (π) was not significantly different between these categories (*P* > 0.054; Tukey’s HSD [Honestly Significant Difference] test), bacteriocins had a higher *d*_N_/*d*_S_ ratio compared with housekeeping and immunity genes (*P* = 0.0066 and *P* = 0.040, respectively; Tukey’s HSD test).

**Table 3:** Molecular Diversity of *blp* Operon and Selected Housekeeping Genes

Gene^a^	Category	Number of Sites	Number of Sequences	Nucleotide Diversity (π)	*d*_N_/*d*_S_
*blpU3*	Unknown	207	5	0.012	2.656
*blpI*	Bacteriocin	147	9	0.023	2.645
*blpJ*	Bacteriocin	226	18	0.012	1.814
*blpQ*	Bacteriocin	201	5	0.010	0.988
*blpO*	Bacteriocin	142	20	0.027	0.974
*blpC*	Regulatory	127	28	0.129	0.895
*pncW*	Bacteriocin	231	19	0.056	0.844
*blpU5*	Unknown	147	8	0.012	0.712
*pncT*	Bacteriocin	192	7	0.009	0.569
*blpU1*	Unknown	414	8	0.014	0.492
*pncG*	Immunity	84	13	0.066	0.476
*blpN*	Bacteriocin	126	19	0.030	0.462
*blpK*	Bacteriocin	213	38	0.040	0.395
*blpM*	Bacteriocin	219	24	0.020	0.360
*blpS*	Regulatory	327	74	0.027	0.352
*pncM*	Immunity	177	22	0.037	0.290
*blpX*	Immunity	357	23	0.023	0.242
*blpH*	Regulatory	1,308	157	0.086	0.206
*blpZ*	Immunity	204	35	0.055	0.199
*pncP*	Immunity	495	128	0.018	0.190
*blpT*	Regulatory	297	53	0.024	0.181
*blpL*	Immunity	222	68	0.068	0.153
*blpP*	Immunity	147	13	0.017	0.152
*aroE*	Housekeeping gene	852	121	0.017	0.151
*blpY*	Immunity	687	127	0.070	0.137
*blp*^b^	Regulatory	1,359	177	0.027	0.137
*gyrA*	Housekeeping gene	2,559	190	0.004	0.117
*blpU4*	Unknown	114	5	0.014	0.092
*xpt*	Housekeeping gene	609	108	0.017	0.090
*blpA*^b^	Regulatory	2,151	74	0.015	0.083
*gdhA*	Housekeeping gene	1,344	161	0.018	0.071
*blpR*	Regulatory	666	114	0.049	0.064
*glkA*	Housekeeping gene	975	141	0.020	0.061
*ddlA*	Housekeeping gene	1,041	189	0.042	0.053
*rpoD*	Housekeeping gene	1,161	113	0.009	0.031
*lepB*	Housekeeping gene	654	101	0.020	0.028
*groEL*	Housekeeping gene	1,620	166	0.014	0.028

^a^*blpD*, *blpE*, *blpF*, *blpG*, *blpI2*, *blpU2*, and *blpV* were removed for having less than five unique sequences after trimming.

^b^Full-length *blpA* and *blpB* alleles only.

### Diversity in Potential Ecological Strategies

There are four unambiguous functional classes of genes within the *blp* operon that could mediate interactions among strains. Bacteriocin and immunity genes define the range of killing and susceptibility, respectively. Additionally, the *blp* locus is regulated by the secretion of the BlpC peptide signal that binds to the quorum sensing receptor, BlpH. Because these signals are secreted, they can be potentially detected and bound by both the secreting cells as well as their competing neighbors. The diverse combinations of these four functional classes, together with the allelic diversity within them, define the potential ecological strategy set by which pneumococcal strains can interact and compete via *blp* bacteriocins. Each strain, by extension, expresses only a single strategy from among this total, and the sum of all such strategies in our data set represents the set of realized strategies.

**Fig. 2.—:**
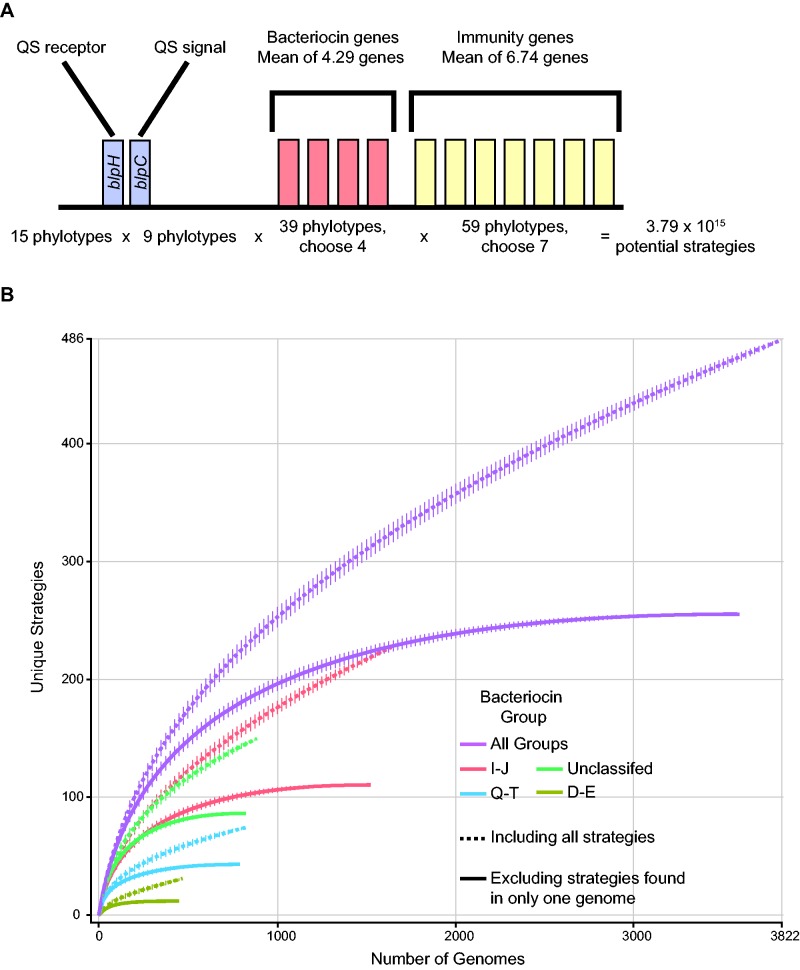
Estimated and sampled ecological strategies in the *blp* operon. (*A*) We reduced the operon to an “average” operon consisting of a histidine kinase quorum sensing receptor (*blpH*), a quorum sensing signals (*blpC*), exactly four bacteriocin genes, and exactly seven immunity genes in order to estimate the number of potential ecological strategies. This average genome was based on frequency data from our genome set, as we detected: 99.1% of genomes containing a single *blpC* gene, with multiple co-occurring *blpC* genes not detected; 99.0% of genomes containing a single *blpH* gene, with multiple co-occurring *blpH* genes not detected; an average of 4.29 putative bacteriocins detected per genome; and an average of 6.74 putative immunity genes detected per genome. We included only phylotypes that were present in at least 0.5% of randomly sampled genomes. (*B*) Rarefaction of ecological strategies found in randomly sampled genomes. Only strains that contained *blpC* and *blpH*, and phylotypes that were present in at least 0.5% of randomly sampled genomes, were included. Error bars indicate standard deviation of 10,000 randomizations.

To estimate the possible number of unique ecological strategies mediated by *blp* operon genes in *S. pneumoniae*, we calculated (using frequency data from our genome set) that an average genome contained a single peptide signal, a single peptide receptor, four bacteriocins, and seven immunity genes (fig. 2*A*). To conduct this analysis, we first examined an admittedly straw-man null hypothesis that assumed phylotypes are functionally distinct and that all genes and phylotypes can assort randomly, without influence of genetic linkage, phylogenetics, or functional constraints. By accounting for the phylotypic variation within these four gene classes (table 1), we calculated that approximately 3.79 × 10^15^ unique ecological strategies, or combinations of phylotypes, were possible (fig. 2A). Based on this estimate, we predicted that it would be necessary to sample an astronomical number of pneumococcal strains to fully characterize the realized diversity at the *blp* locus, and to some approximation this was true. As is evident in figure 2*B*, if we included the 47.5% of unique ecological strategies that appeared in single genomes, we estimated that on average, a new ecological strategy should be found for every 15.4 new strains sampled (assuming that the linear relationship at the end of the rarefaction curve—425 strategies until 486 strategies, *r*^2^ = 0.9996—is realized in perpetuity). In contrast, when we excluded singletons, the rarefaction curve saturated at 255 strategies (fig. 2*B*), providing strong evidence that our data set reflected the prevalent ecological strategies carried within the global pneumococcal population. It is notable that the curve that excluded singleton strategies reached its asymptote after 2,000 genomes, confirming again the necessity of such a large data set. Together, these results suggest there are a relatively small number of common ecological strategies from among all those that are possible, and that global *S. pneumoniae* populations contain a much higher diversity of strategies that occur at extremely low frequencies of less than 5.2 × 10^−^^4^ (as calculated by occurring in single strains out of 3,822 strains with complete strategies; fig. 2B). These data also clarify that the assumption of random assortment between *blp* operon genes and phylotypes is incorrect. Below, we explore different constraints on diversity that contribute to the more limited, although still vast, set of ecological strategies mediated by the *blp* operon, namely constraints imposed by overall operon organization and structure, physical or functional linkage, and phylogeny.

### Constraints on Diversity

#### Operon Structure

**Fig. 3.—:**
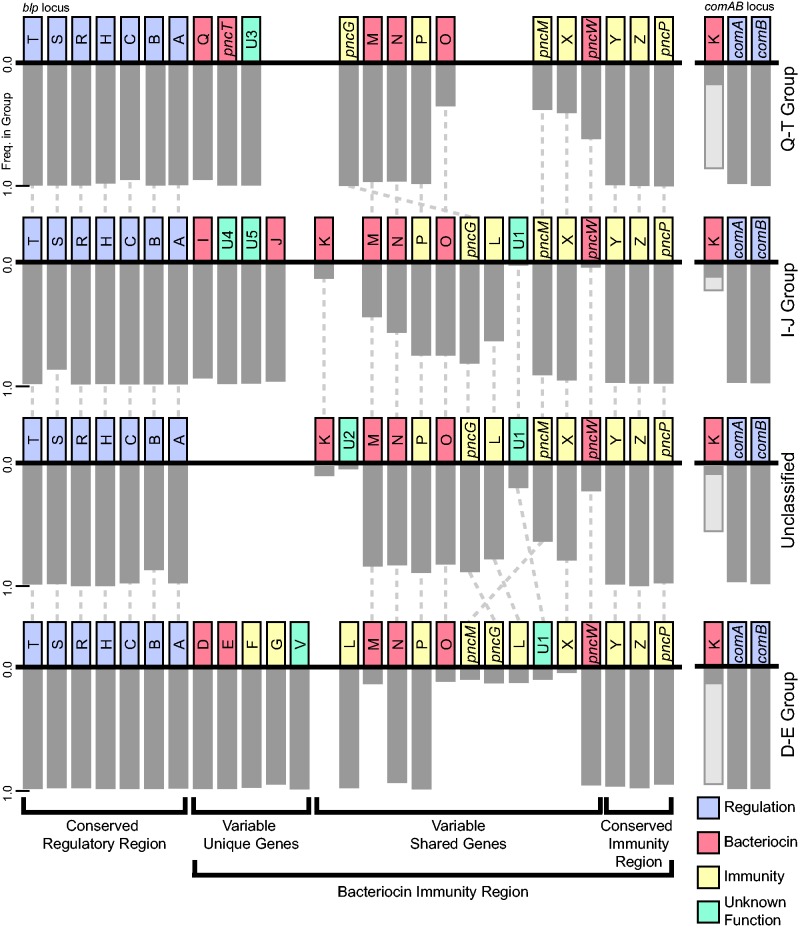
Consensus *blp* operon gene structure across bacteriocin groups. This gene order was compatible with 99.5% of the Q–T group; 94.9% of the I–J group; 93.3% of unclassified genomes; and 99.4% of the D–E group. The frequency of the gene in the group is shown in gray bars; gene function is shown by the color of the gene. Lighter gray bars underneath *blpK* indicate the proportion of genomes with *blpK* in an unknown location. Genes occurring in less than 0.5% of genomes in the bacteriocin group are not shown.

In examining different explanations for the result that there were fewer ecological strategies than expected by gene combinatorics, an initial covariance analysis identified three sets of genes within the *blp* operon in which 1) all genes within each set were found together in genomes at a high frequency (>90%) and 2) genes from different sets were rarely (0.12%) found in the same genome. Based on these criteria, we were able to classify the 4,096 randomly sampled genomes into 3 discrete groups or as “unclassified” (fig. 3): Bacteriocin group Q–T, which included all genomes containing either *blpQ*, *pncT*, or *blpU3* (875 genomes, 93.7% containing all 3 genes); group I–J, which included all genomes containing either *blpI*, *blpJ*, *blpU4*, or *blpU5* (1,742 genomes, 92.0% containing all 4 genes); group D–E, which included all genomes containing either *blpD*, *blpE*, *blpF*, *blpG*, or *blpV* (475 genomes, 94.3% containing all 5 genes); and an unclassified group of genomes that contained none of these bacteriocin group-specific genes (999 genomes). Five genomes (0.12%, termed hybrid genomes) out of 4,096 contained genes for more than one of these bacteriocin groups. The groups had significantly different average number of *blp* bacteriocin and immunity genes (*P* ≤ 6.6 × 10^−^^11^ and *P* ≤ 0.0039 for all putative bacteriocin and immunity genes, respectively; *P* ≤ 1.3 × 10^−^^5^ and *P* ≤ 0.0038 excluding group-specific bacteriocin and immunity genes, respectively; Games–Howell test; supplementary fig. S3, Supplementary Material online), with the exception of an equivalent number of immunity genes in group I–J and in group D–E with group-specific genes (*P* = 0.84). As observed when we analyzed bacteriocin groups together, rarefaction analysis for each bacteriocin group separately again revealed that the number of unique ecological strategies within groups did not saturate with sampling until we removed ecological strategies found in single genomes (fig. 2B). In comparing the bacteriocin groups with the full-genome phylogeny, only the D–E group comprised of a single clade (fig. 1), while the other groups were broadly distributed across the phylogeny.

Gene order was broadly conserved across all genomes, as previous noted (De Saizieu et al. 2000; Reichmann and Hakenbeck 2000; Bogaardt et al. 2015), as well as within bacteriocin groups. Figure 3 shows the consensus gene order for the bacteriocin groups, with each gene order compatible with over 93% of genomes in each group. The *blp* operon can be divided into four regions: The conserved regulatory region; the variable, bacteriocin group-specific genes; variable genes shared across bacteriocin groups; and the conserved immunity region. The last three of these regions is collectively known as the bacteriocin immunity region. In total, 91.4% of genomes carried all seven genes present in the conserved regulatory region, consisting of *blpT*, *blpS*, the response regulator *blpR*, the histidine kinase receptor *blpH*, the quorum sensing signal *blpC*, and ABC transporters *blpB* and *blpA*. Interestingly, only 23.5% of genomes had full-length, potentially functional, versions of *blpB* and *blpA*. However, all examined genomes placed *blpBA* sequences after *blpC*, and here we report the proportion of genomes with copies of *blpB* and *blpA* of any ORF length. The bacteriocin group-specific genes were always located together as a cluster after the conserved regulatory region (fig. 3). Following this region were the variable shared genes; while members of all bacteriocin groups contained these genes, the frequency varied widely across groups (e.g., the proportion of genomes with *blpO* ranged from 10.1% in group D–E to 75.9% in group I–J; fig. 3). Additionally, gene order within this region varied across bacteriocin groups, with *pncG* shifted in group Q–T and *pncM* shifted in group D–E. *blpL* was the only other gene besides *blpK* in which a significant number of genomes contained two copies (1.4% of genomes), each copy with a different phylotype; all except 3 of these 56 genomes were in group D–E. The conserved immunity region, which consists of *blpY*, *blpZ*, and *pncP*, flanked these variable shared genes; these genes were conserved in order and frequency across bacteriocin groups, with 94.9% of genomes containing all three genes.

The location of *blpK* was especially variable between bacteriocin groups. *blpK* was found in two places in group I–J and in unclassified genomes—at the beginning of the *blp* variable shared genes and in the *comAB* locus. The fragmented genome assemblies in these groups prevented determining the exact frequency of *blpK* at either of these loci. However, we found no genome with *blpK* in the *blp* operon for the other 2 bacteriocin groups—group Q–T and group D–E—out of 60 and 35 full genomes, respectively, with complete gene order data. In 95.9% of strains with a copy of *blpK* located in the *comAB* locus (440 out of 459 strains), we found a shared (93.9–100% identity) 65 bp sequence within 100 bp of *blpK*. This same 65 bp sequence was located in 89.0% of genomes (81.5–98.5% identity with the sequence in the *comAB* locus) between *blpA* and the first variable unique gene. The 65 bp sequences, which were identified in our DNA reciprocal BLAST search for transposase-like sequences, translated into the last 15 residues of an IS1381 transposase. Accordingly, this sequence could have or could still provide a homologous sequence for recombination or rearrangement between bacteriocin groups or between the *blp* and the *comAB* loci.

**Fig. 4.—:**
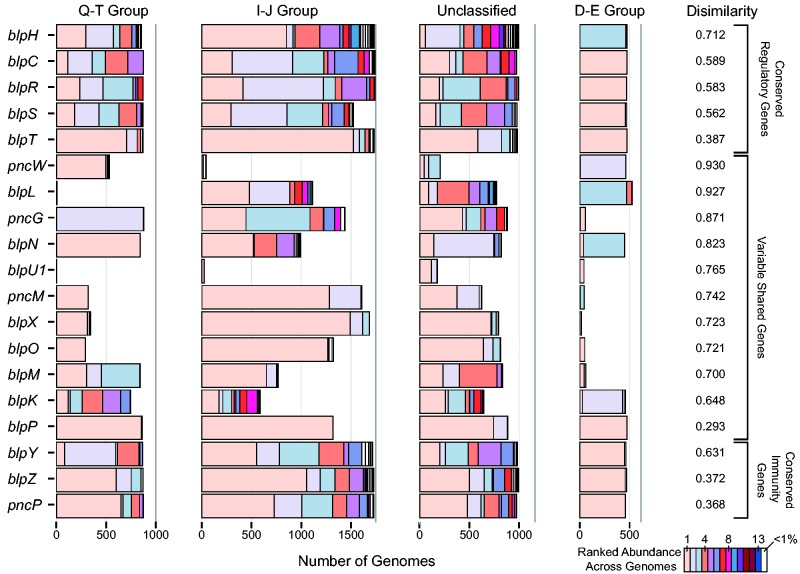
Distribution of phylotypes across bacteriocin groups. For each *blp* operon gene, the number of genomes with each phylotype is shown, with the phylotypes colored by their overall ranked abundance within each gene across all bacteriocin groups. Phylotypes found in less than 1% of randomly sampled genomes are shown in white. Genes are ordered by gene class and Bray–Curtis dissimilarity between bacteriocin groups.

We next examined if phylotypes were biased to particular bacteriocin groups. In direct contrast to the bacteriocin group-specific genes, 81.2% of common phylotypes across all shared genes were not restricted to a single bacteriocin group. However, the quantitative distribution of phylotypes between bacteriocin groups differed significantly for the overwhelming majority of genes (88.8% of gene/bacteriocin group combinations; *P* < 0.0018; pairwise χ^2^ test with Holm–Bonferroni correction, excluding four tests with less than five observed cases; fig. 4). This was especially noticeable for group D–E, which had markedly less diversity across all genes (average Shannon diversity of phylotypes within genes for group D–E = 0.130; 0.733–1.28 for other bacteriocin groups). Variation within genes was concentrated in genes *blpN*, *pncG*, *blpL*, and *pncW*, which all showed a larger amount of phylotypic diversity across groups (Bray–Curtis dissimilarity > 0.82; average across genes = 0.65; standard deviation = 0.19).

#### Allelic Co-occurrence

A potential constraint on realized *blp* diversity is the requirement for functionally interdependent proteins to co-occur within genomes, thereby giving rise to patterns of genic/allelic association that occur more frequently than would be predicted by chance and after correcting for phylogeny. Within the *blp* operon, we predict correlated changes between three *blp* gene categories. First, bacteriocin genes are predicted to have correlated changes with their partner bacteriocin, with which it creates a functional type IIb (two-peptide) bacteriocin outside the cell. Second, bacteriocin genes should have correlated changes with proteins that provide self-immunity to the functional bacteriocin. Third, the BlpC signal molecule is expected to change in concert with its receptor, BlpH. Because few of these associations have been verified empirically, our aim was to identify pairs of proteins whose functional association can be tested in subsequent work, and also to ask whether genes that are predicted to interact do so with high levels of allelic fidelity.

**Fig. 5.—:**
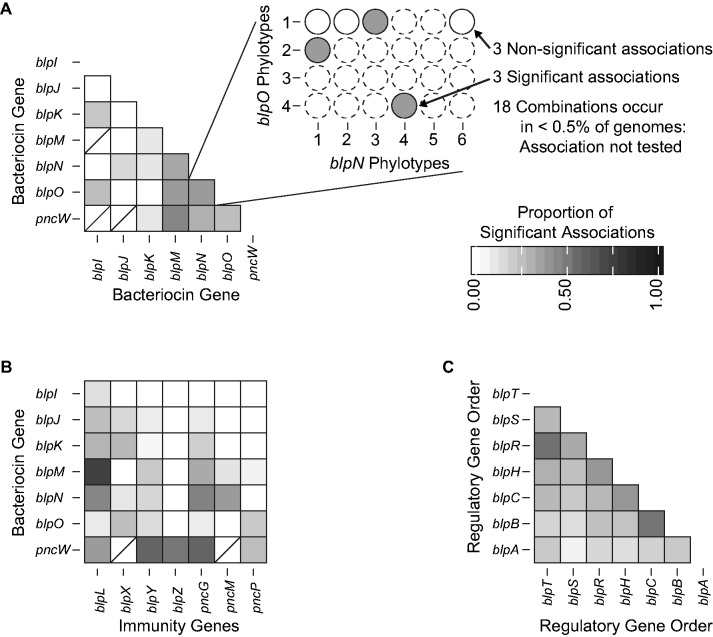
Significant associations between *blp* operon gene phylotypes. Within each pair of genes, the proportion of pairwise phylotypes that had significant associations is shown: (*A*) Between putative bacteriocin genes; (*B*) between putative bacteriocins and immunity genes; and (*C*) between regulatory *blp* operon genes, shown in consensus gene order. Comparisons between phylotypes co-occurring in less than 0.5% of genomes were not tested. Pairs of genes with fewer than three tested phylotype combinations are shown with a slash. Genes with fewer than three phylotypes each occurring in at least 0.5% of genomes are not shown.

In figure 5, we show patterns of association between three categories of *blp* operon genes, which indicate that locus and phylotypic levels of coassociation were highly nonrandom. Importantly, many combinations of phylotypes or genes were extremely rare or absent; for example, 904 out of 1,034 pairs of putative bacteriocin phylotypes in figure 5 co-occurred in less than 0.5% of genomes. However, among the 129 tested pairwise combinations of bacteriocin phylotypes, 26 pairs showed significant associations (fig. 5A). The presence of associations is consistent with the hypothesis that these are type IIb bacteriocins, which require two partner peptides to form functional bacteriocins; however, it is also important to note that these patterns may be partially driven by physical linkage between genes. Finally, this analysis highlights that the fidelity between partner peptides was not absolute. As an example, *blpN* had significant associations with specific phylotypes of *blpJ*, *blpK*, *blpM*, *blpO*, and *pncW*; while experimental evidence has shown that one phylotype of *blpN* formed a functional bacteriocin with one phylotype of *blpM* (Dawid et al. 2007), these additional associations raise the possibility of multiple partner peptides with various *blpN* phylotypes.

In 88 out of 491 tested pairwise combinations of putative bacteriocin and immunity phylotypes, we found significant positive associations that are candidates for immunity against specific bacteriocins (fig. 5B). This includes established and putative immunity genes that have associations with many bacteriocin genes (*blpL*, *blpY*, *blpZ*, and *pncP*, all associated with 1–7 putative bacteriocin genes), as well as newly discovered *pncG* (Bogaardt et al. 2015), which is associated with six putative bacteriocins. As with pairs of *blp* bacteriocin phylotypes, there was not strict fidelity of one-bacteriocin:one-immunity gene, suggesting the possibility that genes for immunity may provide cross-protection against several bacteriocins.

In testing for *blpC*–*blpH* association, we found strong patterns of nonrandom co-occurrence (fig. 5C), as expected. Also, consistent with the effects of physical linkage, we observed that associations between genes within the entire regulatory region scale with gene proximity. Specifically, regulatory *blp* operon genes physically next to each other had a higher proportion of significant associations than other regulatory *blp* operon genes (average proportion significant association for neighboring genes = 0.465; nonneighboring genes = 0.294; *P* = 0.024; Mann–Whitney test).

#### Phylogenetic Constraints

**Fig. 6.—:**
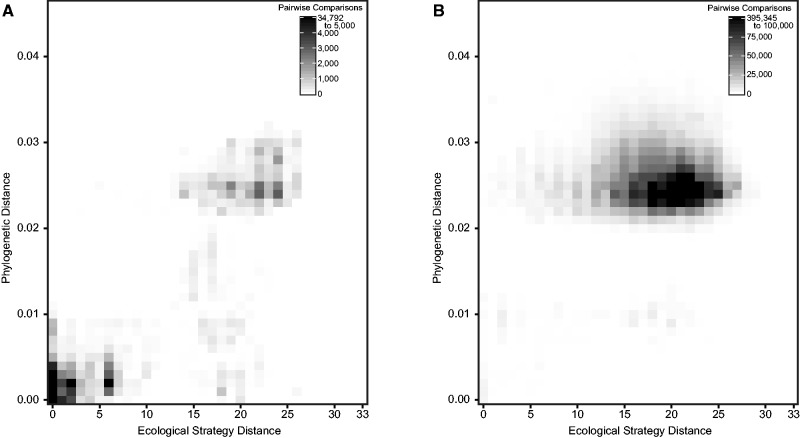
Distribution of pairwise strain comparisons across phylogenetic and ecological strategy distance. For strains that were placed into clonal complexes, we calculated the pairwise distance along the phylogenetic tree and the pairwise distance between ecological strategies (e.g., the number of different gene-phylotypes for *blpH*, *blpC*, putative bacteriocins, and immunity genes) of (*A*) strains belonging to the same clonal complex and (*B*) strains belonging to different clonal complexes. The axes divisions are identical for each graph, although the scales differ. Figure 6*A* has 14 divisions with over 5,000 pairwise comparisons, while figure 6*B* has 24 divisions with over 100,000 pairwise comparisons.

As the D–E group comprised a single clade (fig. 1), we next examined if phylogeny constrained the number of realized ecological strategies. We first classified genomes into clonal complexes based on MLST, and calculated the effective distance between ecological strategies by quantifying the number of differences between pairs of strains in phylotypes for *blpC*, *blpH*, bacteriocin genes, and immunity genes. The results in figure 6 show that there is a strong influence of phylogenetic distance on ecological distance, and that strains within clonal complexes were more similar to one another with respect to ecological strategies than they were to strains in different clonal complexes, whereas strains in the same clonal complex had an average distance of 6.74 between potential ecological strategies (fig. 6A), and strains in different clonal complexes had an average ecological strategy distance of 18.76 (fig. 6B; *P* < 10^−^^99^, Mann–Whitney test). Interestingly, a sizable minority of strain pairwise comparisons (28.5%) within the same clonal complex differed as much or more in ecological strategies than the upper 95% of all comparisons across clonal complexes (ecological strategy distance ≥ 11), as seen in figure 6A. Of these, 87.1% pairwise strain comparisons were quite phylogenetically diverged (to the same degree as across clonal complex comparisons with a phylogenetic distance greater than 0.01), perhaps reflecting the fact that clonal complexes are themselves imperfect representations of diversity across the genome (Turner et al. 2007). However, the remaining 12.9% were highly related, and in 77.5% of these comparisons, the ecological divergence resulted from switches in bacteriocin groups, which are likely due to recombination of the entire operon. Further studies are needed to analyze if these recombinants were positively selected, or if this variation was a result of neutral drift.

## Discussion

### Comprehensive Sampling Revealed Extensive Genic and Allelic Diversity

Bacteriocins may be crucial regulators of bacterial community dynamics, yet there is little understanding of the *blp* operon that mediates these interactions in *S. pneumoniae*. Here we used a comprehensive bioinformatics approach to understand diversity in this operon. At every level of classification, we found an unexpectedly large amount of variation: In the number of putative bacteriocin and immunity genes (supplementary fig. S3, Supplementary Material online), the presence or absence of specific *blp* operon genes (fig. 3), gene order (fig. 3), and the tremendous breadth of phylotypic variants (fig. 4)—all of which resulted in an exceptionally large diversity of *blp* operon arrangements. Yet despite the fact that thousands of *S. pneumoniae* genomes were required to reveal both the genic composition as well as the full allelic diversity of the genes in this operon, rarefaction analysis indicated that although we identified most of the diversity, more remains to be discovered (fig. 2*B* and supplementary fig. S2, Supplementary Material online). Nearly 50% of the combinations of all *blp* ecological strategies were unique (fig. 2*B*), suggesting that new arrangements emerge frequently; moreover, it was clear that the only way to fully understand the diversity of this locus was by taking advantage of the vast sequencing data sets of this species. In so doing, we identified the novel putative bacteriocin gene *blpI2* and five potential genes *blpU1*–*blpU5.* Additionally, we found nine common (0.5% or greater) variants of the BlpC signal molecule, compared with the previously reported four to six common BlpC mature peptides (Bogaardt et al. 2015; Pinchas et al. 2015). These analyses extend results from another recent examination of the *blp* operon (Bogaardt et al. 2015), which also reported on the astounding diversity in this operon as well as organized the previously chaotic *blp* operon gene nomenclature (Bogaardt et al. 2015).

### Possible Constraints on the Number of Realized Ecological Strategies

We began with the naive null hypothesis that assumed phylotypes are functionally distinct (which they are not designed to indicate) and that all phylotypes can freely associate with one another. As described in figure 2, this straw-man hypothesis then produced an estimate of approximately 10^15^ unique ecological strategies. Because each arrangement could potentially specify a distinct mode of signaling, killing, and susceptibility, each represents a potential strategy by which strains interact and compete with one another. Yet instead of 10^15^ possible strategies, we found significantly fewer, with rarefaction analysis (excluding singletons) saturating at around 250 combinations (fig. 2). Our analyses provide several explanations for why the data do not conform to these extreme, simplifying assumptions. First, we showed that genomes could be classified into distinct groups that contain unique sets of genes (fig. 3). These “variable unique genes” had a conserved orientation in the *blp* operon; however, at present little is known of either the specific functional relevance of these genes or the factors that lead to their patterns of association. Interestingly, each group, with the exception of the D–E group, was broadly distributed across the strain phylogeny (fig. 1). This is consistent with the idea that horizontal gene transfer had mobilized genes within this region to unrelated strains. However, although this could explain the phylogenetic dispersion of bacteriocin groups, it would not explain the associations between the genes themselves. Nor does it explain other group-specific differences in putative bacteriocin and immunity gene number and the differential presence/absence of specific genes in the *blp* operon (fig. 3). Identifying the functional reasons, if any, for these associations remains an important area for further investigation.

A second type of constraint that would limit the number of realized *blp* strategies is physical or functional linkage interactions among genes and phylotypes within the *blp* locus: BlpC binds to its cognate receptor, BlpH; type IIb bacteriocins are believed to act co-ordinately in pairs; and cells expressing specific bacteriocins must be protected from suicide by immunity. As expected, given these presumed associations, we found strong correlations between specific phylotypes of *blp* operon genes (fig. 5). Equally informative was the fact that many phylotypes were never found together, and interestingly this differed across various regions of the operon. For example, there were extensive associations among genes regulating *blp* activation, in addition to nearly complete conservation of gene order in this region; however, there was markedly less coassociation between putative bacteriocins or between bacteriocins and immunity. Each of these associations, particularly for cases not influenced by physical linkage, is suggestive of functional relationships that can be examined experimentally.

Although these patterns of coassociation can be interpreted as a mechanism constraining overall diversity in the *blp* operon, it is also important to realize that the coassociations can also reveal a potential cause of functional diversification. This is most easily seen for the bacteriocins that are believed to function as two-peptide pairs. One possibility is that these pairs are highly precise, showing complete fidelity at both the genic and phylotypic levels. Alternatively, if different phylotypic variants retain activity with distinct partners, as recently described in a two-component lantibiotic system (Zhao and van der Donk 2016), then this could potentially expand the target range of each “class” of type IIb bacteriocins. For example, BlpM and BlpN have been shown experimentally to interact to generate lethal activity to the target cell (Dawid et al. 2007); yet in the D–E group, the frequency of strains containing *blpM* was only 12.2% compared with 94.3% containing *blpN* (fig. 3), suggesting either a very slow decay rate for ineffective bacteriocin peptides or the possibility that these genes combine with other peptides to become active. Additionally, within each of these genes there was marked phylotypic diversity. *blpM* had 5 common phylotypes and *blpN* had 6, leading to 30 potential combinations; of these, only 9 were realized, consistent with the ideas of functional constraints or genetic linkage. At the same time, many phylotypes occurred with multiple partners, suggesting that either strict fidelity is not required to retain function or alternatively that tight associations have not been selected for; such infidelity between bacteriocin peptide partners has not been described previously. Regulatory genes showed a similar lack of strict phylotypic association (fig. 5*C*); however, it is important to note that there was evidence that these associations could be partially caused by physical gene linkage, a possibility that also holds true for bacteriocin–bacteriocin associations (fig. 5*A*).

A potential epistatic interaction could exist between the *blpAB* ABC transporter and both bacteriocins and the quorum sensing signal BlpC, as nonfunctional BlpAB would not export these peptides. Although only 23.5% of strains had intact *blpAB*, the competence-related ABC transporter ComAB has been shown to export BlpC (Kjos et al. 2016). The export of bacteriocins by ComAB has not been examined, but strains with interrupted *blpAB* had on average only 0.14 fewer putative *blp* bacteriocins than strains with intact *blpAB* (Kjos et al. 2016). This suggests that another ABC transporter can export *blp* bacteriocins, as there were few signs of evolutionary decay within putative *blp* bacteriocin genes.

Phylogenetic conservation also provides an explanation for the limited number of observed ecological strategies in our sequenced genomes, as reflected in the significant similarity among the ecological strategies within clonal complexes (fig. 6). It is interesting, however, that variation within clonal complexes is still present, indicating that even closely related strains may frequently switch bacteriocin groups or diversify via recombination at this locus; further investigation will be needed to examine if these changes among related ST are driven by neutral or selective factors.

### Mechanisms of Diversification

Although various constraints undoubtedly limit the number of realized *blp* ecological strategies, 255 moderately common strategies still bear explaining; what creates and maintains these strategies? From an ecological and evolutionary standpoint, we hypothesize that antagonistic relationships between competing strains in the nasopharynx select for bacteriocin diversity. Interference competition, in which individuals expend resources to actively inhibit others’ growth, leads to a winner-takes-all system of low diversity in a well-mixed environment. However, interference competition with spatial structure has the potential to increase global diversity by creating several locally optimal ecological strategies but with no single, globally best ecological strategy (Riley and Gordon 1999; Riley and Wertz 2002; Abrudan et al. 2012; Hawlena et al. 2012). Assuming that bacteriocin production and immunity come with fitness costs, trade-offs between killing, immunity, and faster growth can lead to nontransitive dynamics that promote and preserve diversity across local sites in silico (Czárán et al. 2002), in vitro (Kerr et al. 2002; Majeed et al. 2011), and in vivo (Kirkup and Riley 2004; Bakkal et al. 2010). Novel ecological strategies are constantly selected for, as the frequency of specific, local strategies is ever-changing. This would explain the variation seen at three specific levels in the *blp* operon: In the rapid molecular evolution of bacteriocins (table 3); in the diversity of phylotypes within genes (fig. 4); and in the changing gene composition of the *blp* locus (fig. 3). The lower variation within and between genes in the D–E group compared with other bacteriocin groups (figs. 3 and 4) may suggest that a different evolutionary force was acting on this bacteriocin group; while the D–E group was tightly clustered (fig. 1), more investigation is needed to determine if this is evidence of an expanding or a declining lineage.

At a more mechanistic level, different factors could explain the diversification of the *blp* operon, in turn helping to explain the vast (and possibly limitless) number of unique ecological strategies. First, the mutation rate of individual genes could be high, although there is no evidence to support this possibility. Second, and more likely, a key driver of allelic and genic diversity at the *blp* locus may be recombination, as *S. pneumoniae* is naturally transformable with high rates of horizontal gene transfer occurring in both liquid and surface associated communities (Johnston et al. 2014). For example, the first 12 residues of 2 *blpN* variants have recombined with *blpM* and *blpK* (table 2). Recombination can explain the presence of two distinct presumed leader sequences within *blpN* and *blpK* (supplementary table S5, Supplementary Material online) as well the *blpM* variant found in 1.1% of genomes (table 2), which encodes for two bacteriocin peptides within one gene. As noted above, the presence of similar, transposase-derived sequences following *blpA* and before *blpK* is a potential region of homologous recombination that can underlie the transfer of variable unique genes between strains, as well as the intragenomic mobility of *blpK* itself. In contrast to other genes in the operon, *blpK* is located in two positions, either within the canonical operon or alternatively adjacent to *comAB.* The recent discovery of a circular bacteriocin next to *comAB* (Bogaardt et al. 2015) and experimental evidence demonstrating the ability of ComAB to export BlpC (Kjos et al. 2016) suggests a strong relationship between *comAB* and bacteriocin production.

Our analyses have uncovered unprecedented diversity at the *blp* operon and highlighted numerous questions that can be examined empirically. This is important for understanding the role of these toxins in interference competition and pneumococcal strain prevalence, but also as a way to potentially identify new bacteriocins and bacteriocin targets that may be of clinical value. This holds true for classical type IIb bacteriocins but also for new bacteriocins that fall into different functional types. Toward that end, it is notable that the number of putative bacteriocin genes per genomes did not peak at even numbers (supplementary fig. S3, Supplementary Material online), which would be expected if *blp* bacteriocin genes were always found with their dedicated partner when forming a functional type IIb bacteriocin. Additionally, there were significantly higher numbers of immunity genes than putative bacteriocin genes, especially after considering that bacteriocin genes are predicted to function in pairs; this suggests that there is not a one-to-one relationship between bacteriocin and immunity genes. Finally, the relationship between the CAAX amino protease immunity genes (e.g., *pncP*, *blpG*, and *blpY*; Pei and Grishin 2001; Kjos et al. 2010, 2011; Bogaardt et al. 2015) and the shorter, membrane protein immunity genes (e.g., *blpL*, *blpX*, *pncG*, *pncM*, and *blpZ*) is unclear and needs to be elucidated to fully understand how immunity protects against specific bacteriocins.

In summary, thousands of *S. pneumoniae* genomes were required to saturate the diversity at the *blp* locus. Although trillions of ecological strategies are possible within this diversity, hundreds are instead realized. The reason for this discrepancy is that bacteriocins are found in discrete groups, that correlations between functionally or physically linked genes limit combinations among genes and phylotypes, and that clonally related genotypes share highly similar potential *blp* ecological strategies. Diversity lies mainly in the presence or absence of *blp* operon genes that then form extremely rare ecological strategies found in less than 5.2 × 10^−^^4^ of genomes (i.e., singleton strategies in our genome set). The *blp* system in *S. pneumoniae* provides an opportunity to further study interference competition in which hundreds of millions of strategies can potentially exist.

## Supplementary Material

Supplementary tables S1–S5 and figures S1–S3 are available at *Genome Biology and Evolution online* (http://www.gbe.oxfordjournals.org/).
